# Fabrication of Rigid Isocyanate-Based Polyimide Foam Achieved Excellent Use Safety via Synergy between Expandable Graphite and Phosphorus-Containing Polyol

**DOI:** 10.3390/polym15061381

**Published:** 2023-03-10

**Authors:** Junhe Zhang, Yingze Liu, Xin Fu, Ting Wang, Gaohui Sun, Xu Zhang, Shihui Han

**Affiliations:** 1 College of Materials Science and Chemical Engineering, Harbin Engineering University, Harbin 150001, China; 2 Wuhan Second Ship Design and Research Institute, Wuhan 430064, China; 3 School of Safety Engineering, Shenyang Aerospace University, Shenyang 110136, China

**Keywords:** rigid polyimide foams, expandable graphite, phosphorus-containing polyol, flame retardancy, toxic fume

## Abstract

For the advantages of low cost, excellent thermal insulation, and sound absorption properties, the rigid isocyanate-based polyimide foam (RPIF) presents great application prospects as a building insulation material. However, its inflammability and the accompanying toxic fumes create huge safety hazard. In this paper, reactive phosphate-containing polyol (PPCP) is synthesized and employed with expandable graphite (EG) to obtain RPIF with excellent use safety. EG can be considered as an ideal partner for PPCP to weaken the drawbacks in toxic fume release. Limiting oxygen index (LOI), cone calorimeter test (CCT), and toxic gas results show that the combination of PPCP and EG can synergistically enhance flame retardancy and the use safety of RPIF owing to the unique structure of a dense char layer possessing a flame barrier and toxic gas adsorption effects. When EG and PPCP are simultaneously applied to the RPIF system, the higher EG dosage will bring higher positive synergistic effects in the use safety of RPIF. The most preferred ratio of EG and PPCP is 2:1 (RPIF-10-5) in this study; RPIF-10-5 shows the highest LOI, low CCT results and specific optical density of smoke, and low HCN concentration. This design and the findings are of great significance to improving the application of RPIF.

## 1. Introduction

Polyimide foam (PIF) has been widely using in marine, aviation, and other fields, owing to its excellent high–low-temperature tolerance, flame retardancy, thermal stability, and outstanding use safety [1,2,3,4,5,6]. At present, the production route of PIF using aromatic anhydride and amine through microwave foam molding is most mature [7]. However, the high production cost, low yield, complex production process, long production cycle, and high market price of PIF are the most serious drawbacks of this route, and limit application [8,9].

Isocyanate-based polyimide foam (IBPIF) using isocyanate and aromatic anhydride or its derivatives as raw materials is a new kind of PIF and can be easily produced by the liquid phase foam route as polyurethane foam [10]. The first advantage of IBPIF is that cheaper aromatic anhydride and isocyanate can be used, which greatly reduces cost [8,11]. Secondly, the production process only includes slurry synthesis, free foaming, and high-temperature curing, and so it greatly simplifies the production route and reduces production costs [12]. These two factors will establish the foundation of PIF used in civil fields such as buildings’ and refrigeration thermal insulation. However, the density of common IBPIF is only about 5~15 kg/m^3^ and its compression strength is only about 10~30 kPa, so it is usually used as a flexible foam, and its strength is far below the requirement of ISO 4898-2018 [13]. Fortunately, when a closed mold process is added during the foaming process, IBPIF possessing higher strength and hardness can be easily obtained, and is named rigid IBPIF (RPIF) [14,15]. Moreover, RPIF still possesses excellent temperature tolerance (−100~260 °C), thermal insulation (λ = 0.03~0.05 W/(m·K)), and sound absorption properties [16,17,18].

The viscosity and ratio of foaming slurries are major factors determining the cellular structure and resin structure of RPIF, respectively. Our previous studies indicate that IBPIF can possess uniform cells when the ratio of carboxylic acid ester precursor solution (with 45 wt. %) to isocyanate is between 1:1 and 5:1. However, the by-products (including polyurea and self-polymerizing products) of isocyanate in RPIF are highly flammable, while a large amount of toxic smoke (including HCN and CO) is generated during the combustion process [19,20,21]. Therefore, in order to solve the safety hazards and ensure the use safety of RPIF, problems including flammability, strong smoke release, and toxic fume generation have become the research focuses of RPIF for its long-term development.

The application of flame retardants is universally regarded as a simple, efficient, and economical choice to enhance the use safety of RPIF [22,23,24]. Phosphorus-containing liquid flame retardants have been widely used in polymer products given their high flame-retardant efficiency and low price, among other features [25,26,27]. As a highly efficient flame retardant, Feng et al. found that the PO radical generated from dimethyl methylphosphonate (DMMP) could quench H and OH radicals during the combustion of polymers and inhibit their continuous burning [28]. Meanwhile, phosphoric acid derivatives, including polyphosphoric acid and pyrophosphate, could achieve the effective catalytic carbonization of polymers and promote the formation of a char layer to further isolate the flame [29].

Xu et al. demonstrated that the limiting oxygen index (LOI) of rigid polyurethane foam (RPUF) with a dosage of tris(1-chloro-2-propyl) phosphate of only 20 wt. % could reach 24.8%, which is increased by 26.5% compared with that of pure RPUF. The total heat release (THR) decreased from 41.9 MJ/m^2^ to 27.8 MJ/m^2^ [30]. In addition, Feng et al. found that at 10 wt. % DMMP, the LOI of RPUF increased from 19.2% to 24.6%, and the peak of heat release rate (PHRR) decreased from 317 to 230 kW/m^2^ [31]. However, in contrast to RPUF, the process of preparing RPIF includes a curing process at about 200 °C. Unfortunately, phosphorus liquid flame retardants usually have a low boiling point, and they violently evaporate during the curing process of IBPIF [11]. This reduces the actual flame retardant effect of phosphorus-containing liquid flame retardants. Moreover, only physical mixing with the matrix occurs, and the continuous free emission into the environment is also a source of pollution [32]. Thus, for the use of phosphorus-containing liquid flame retardants in RPIF, there is an urgent need to achieve their functionalization and effective chemical bonding with matrix resin.

Regarding the functionalization, Rao et al. successfully synthesized phosphorus-containing polyol (PPCP) using phenyl-phosphonic dichloride and ethylene glycol [33]. Zhang et al. opportunely synthesized PPCP using DMMP and 1,6-hexanediol by a transesterification reaction [34]. Then, a series PPCP was used in the preparation of flame-retardant polyurethane foam owing to the chemical reaction between hydroxyl and isocyanate groups. For IBPIF, because it also applies isocyanate as a raw material, PPCP will also form effective chemical bonds with matrix resin. The chemical bonding will directly address the evaporation of phosphorus-containing liquid flame retardants during the high-temperature curing process of IBPIF. On the basis of this approach and to further increase the flame retardancy of IBPIF, this work seeks to employ a cheap and efficient PPCP as a phosphorus-containing liquid flame retardant to substitute the common unreactive phosphorus-containing liquid flame retardant used in IBPIF. The influences and efficacy of PPCP on flame retardancy are systematically studied.

However, numerous studies have indicated that the incorporation of a phosphorus-containing structure in polymer materials induces more severe smoke release and toxic fume generation. In fact, the problem of the smoke toxicity of polymer materials during burning is the most dangerous safety issue, and is the primary reason for deaths in fires [35].

Expandable graphite (EG) is an intumescent flame retardant; it is non-toxic, environmental, and cheap [36]. For polymeric foams, EG possesses more significant smoke-suppressant effects than its flame-retardant effects, owing to the formation of porous and worm-like graphitic carbon [37,38,39]. Thus, EG has always been regarded as an ideal smoke-suppressant. Zhang et al. reported that only a 10 wt. % dosage of EG in RPUF could reduce total smoke production (TSP) from 4.13 to 2.28 m^2^ [40].

In this paper, considering the disadvantages of using PPCP alone regarding smoke and toxic fume release, it is here combined with EG to improve the use safety of RPIF. This study systematically investigated the synergy between EG and PPCP regarding the flame retardancy, smoke release, and toxic fume generation performances of RPIF. The corresponding mechanism is also proposed. Meanwhile, in order to produce safe and cheap RPIF to widen its application, DMMP, which is a phosphorus-containing liquid flame retardant possessing high flame retardant efficiency and a low price, is selected to synthesize PPCP.

## 2. Materials and Methods

### 2.1. Materials

Pyromellitic dianhydride (PMDA, >99.5%) was provided by Nanjing Lonza Co., Ltd (Nanjing, China). Methanol (AR), *N*,*N*-dimethyl formamide (DMF, AR), and triethanolamine (TEA, AR) were purchased from Tianjin Fuyu Fine Chenical Co., Ltd (Tianjin, China). Dibutyltin dilaurate (T12, CP) was supplied by Tianjin Guangfu Fine Chemical Institute (Tianjin, China). Polysiloxane-polyether copolymer (AK8805, >99.8%) was acquired from Nanjing Maysta New Material Co., Ltd (Nanjing, China). Ethylene glycol and monobutyltin oxide (MO) were obtained from Sinopharm Chemical Reagent Co., Ltd (Shanghai, China). Dabco-33 was synthesized as in a previous work [8]. Polymethylene polyphenylene isocyanate (PAPI, ≥99.8%) was supplied by Wanhua Chemical Group Co.,Ltd (Yantai, China). DMMP was purchased from Beijing Jintong Letai Chemical Products Co., Ltd (Beijing, China). EG (80 mesh) was supplied by Qingdao Yanhai Carbon Material Co., Ltd (Qingdao, China).

### 2.2. Samples Preparation

#### 2.2.1. Synthesis of PPCP

**Scheme 1:**
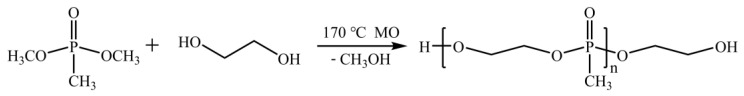
Synthesis route of PPCP.

PPCP was synthesized via the reaction in Scheme 1. Ethylene glycol was selected to react with DMMP to synthesize the target product for its high reactivity and low steric hindrance; the generated methanol could also be directly distilled from the reaction system. DMMP (1.00 mol, 124.08 g) and monobutyltin oxide (0.37 g) were mixed evenly. When the temperature of the mixture reached 170 °C, ethylene glycol (1.10 mol, 68.27 g) was added drop by drop. The reaction was executed 4 h at 170 °C. After a vacuum distillation process for unreacted raw materials was undertaken at 120 °C, light yellow PPCP was derived.

#### 2.2.2. Preparation of Precursor Solution

Firstly, a mixture of 28.90 g DMF and 17.45 g PMDA was heated to 55 °C, then 5.00 g of methanol was dropped in 5 min. After 15 min, a transparent solution was derived, referred to as the precursor solution.

#### 2.2.3. Composition of Slurry

**Scheme 2:**
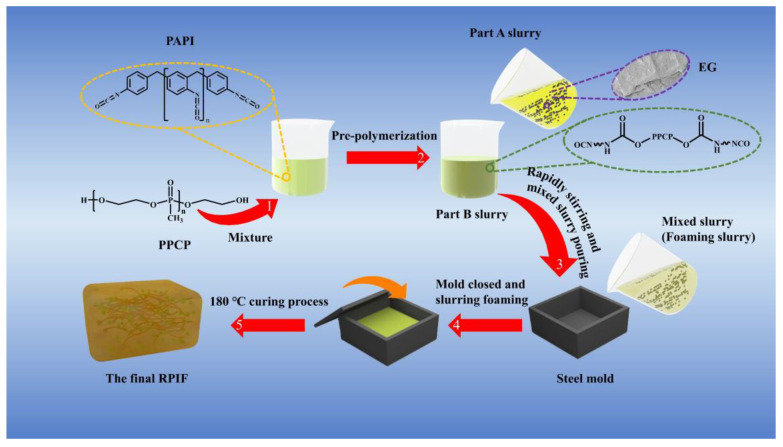
Schematic diagram of RPIF preparation process.

In total, 23.7 g precursor solution, dibutyltin dilaurate (0.3 g), triethanolamine (1.4 g), 0.5 g Dabco-33, polysiloxane-polyether copolymer (3.4 g), 3.3 g deionized water, and pre-weighed EG were mixed evenly. The homogeneous mixture was referred to as Part A slurry. A detailed diagram of the process of RPIF preparation is shown in Scheme 2. The pre-weighed PPCP was firstly mixed with 40.9 g PAPI, and the mixture was named Part B slurry; this step is described as the number 1 step in Scheme 2.

The detailed dosages of EG and PPCP in the corresponding slurry of each RPIF are listed in Table 1. In all cases, the presented mass weight percent of EG and PPCP is the mass ratio of their dosage to the PAPI dosage. For convenience, the corresponding slurry is directly named Part AX or Part BX. X stands for the dosage of EG or PPCP. For example, when the dosage of PPCP added to PAPI was 15 wt. %, the slurry is referred to as Part B15, so pure PAPI is referred to as Part B0, and the Part A slurry containing no EG is referred to as Part A0.

#### 2.2.4. Preparation of RPIF

Before used, the Part A and Part B slurries were all cooled to −5 °C; the pre-polymerization between PPCP and PAPI was also completed in this process, corresponding to step 2 in Scheme 2. Then, the Part A slurry was immediately poured into Part B slurry followed by mechanical stirring (1500 rpm) for about 15 s. Subsequently, the mixed slurry (foaming slurry) was transferred into an open steel mold (15 × 15 × 7 cm^3^), and the top cover was quickly fixed on. These operations correspond to step 3 and 4 in Scheme 2. After 20 min, the mold was transferred into an oven at 180 °C and heated for about 2 h. Finally, the mold was taken apart and RPIF was obtained, corresponding to step 5 in Scheme 2.

**Scheme 3:**
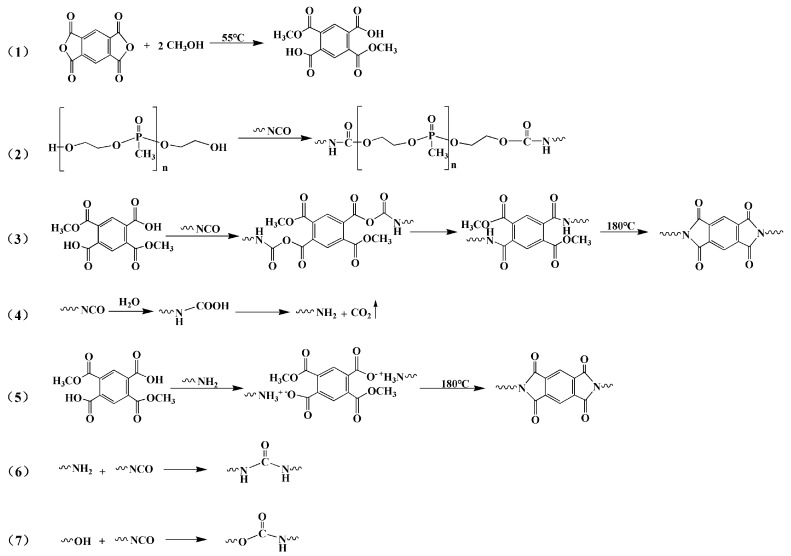
The chemical reactions involved in the production of RPIF.

The chemical reactions involved in the production of RPIF are listed in Scheme 3 [8]. The esterification reaction between the anhydride and methanol led to the formation of the precursor and is listed as (1) in Scheme 3; the chemical reaction between PAPI and PPCP leading to the polyurethane (slurry B) is listed as (2) in Scheme 3. The reaction between the isocyanate group and water leading to the generation of carbon dioxide, the foaming process and the formation of cells is listed as (4) in Scheme 3. The reactions leading to the final polyimide (PI) are listed as (3) and (5) in Scheme 3. The isocyanate group and amino group generated from reaction (4) in Scheme 3 can all react with the precursor generated from reaction (1) in Scheme 3 to form PI depending on the high temperature conditions during the curing process. Reactions (6) and (7) in Scheme 3 involve the formation of other components possessing an amide structure (-COR-NH-). The reaction between the isocyanate group and amino group generated in reaction (4) forms polyurea, while the reaction between the isocyanate group and hydroxyl group in other agents forms polyurethane.

Regarding the serial numbers of different RPIF, because EG was added into the Part A slurry while PPCP was added into the Part B slurry, the general formula has been marked as RPIF-E-P. E stands for the dosage of EG, while P stands for the dosage of PPCP. For example, when Part A10 and Part B15 were used to generate RPIF, its detailed serial number was RPIF-10-15. The RPIF-E-0 series, including RPIF-0-0, RPIF-5-0, RPIF-10-0, and RPIF-15-0, which contained no PPCP, is used to discuss the effect of EG dosage alone on the safety of RPIF. Similarly, the RPIF-0-P series is used to discuss the effect of PPCP dosage alone on the use safety of RPIF.

### 2.3. Characterization

Fourier Transform Infrared (FT-IR) spectra were obtained using a PerkinElmer Spectrum 100 spectrometer (PerkinElmer, Waltham, MA, USA) in a range from 500 to 4000 cm^−1^ for 8 scans. The apparent density of RPIF was determined in accordance with ISO845: 2006 using 100 mm × 100 mm × 20 mm specimens, and the final value was the average of three samples. An Apreo S Scanning Electron Microscope (SEM)–Energy Dispersive Spectrometer (EDS) (Thermo Fisher Scientific, Waltham, MA, USA) was used to obtain the cellular structure morphology and perform the elemental analysis of RPIF. The LOI of RPIF was obtained with a JF-3 oxygen index tester (Nanjing Jiangning Analytical Instrument Factory, Nanjing, China) according to ISO 4589-1: 2017. The size of samples was 200 mm × 10 mm × 10 mm, and the final value was the average of three samples. Thermogravimetric analysis (TGA) was performed via a TA Instruments Thermometric Analyzer Model Q50 (TA Instruments, New Castle, DE, USA) at a heating rate of 20 °C/min under nitrogen atmosphere. A cone calorimeter test (CCT) was performed with an FFT007 cone calorimeter (Fire Testing Technology Limited, West Sussex, UK) at 35 kW/m^2^ heat flux according to ISO 5660-1: 2015; the dimension of the sample was 100 mm × 100 mm × 20 mm. The specific optical density of smoke (Ds) indicating the optical property of materials under specified test conditions implemented to produce smoke density was assessed in an FTT test chamber (Fire Testing Technology Limited, West Sussex, UK) with a pilot flame at heat flux of 25 kW/m^2^ according to ISO 5659-2: 2017. The size of specimens was 75 mm × 75 mm × 10 mm and the test time was 1200 s. The toxic fume generation test was performed in an NES-713 smoke toxicity test chamber (PHINIX, Suzhou, China) using methane as the ignition gas according to NES713; the hydrogen cyanide (HCN) concentration was measured in this research with a GASTEC detection tube, and the detection range of the HCN GASTEC detection tube was 2.5~60 ppm. The mass of every tested sample was 1g and corresponded to about 32 cm^3^. X-ray photoelectron spectroscopy (XPS) (PHI 5700 ESCA, PerkinElmer, Waltham, MA, US) with monochromatic Al-Kα radiation as the X-ray source (hn = 1486.6 eV) was employed to characterize the bonding structure of the residual char layer of RPIF after CCT. The high-resolution test step was 0.05 eV. A LabRAM HR800 Raman spectrometer (Horiba Jobin-Yvon, Paris, France) was used to obtain the C element spectra in the residual char layer of RPIF after CCT in a range from 2000 to 800 cm^−1^.

## 3. Results and Discussion

### 3.1. FT-IR Characterization of PPCP and Partial Part B Slurry

**Figure 1:**
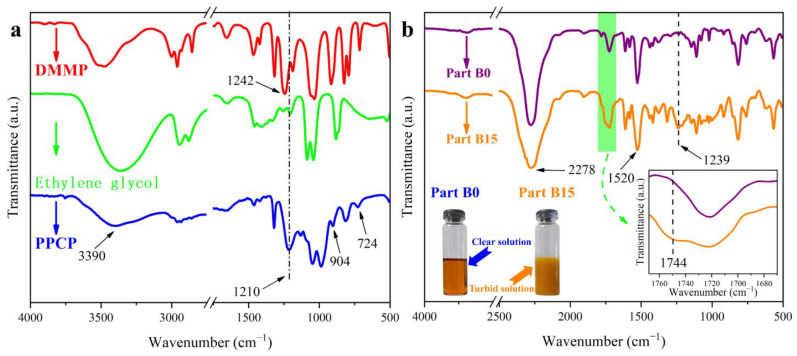
(**a**) FT-IR curves of DMMP, ethylene glycol, and PPCP. (**b**) Digital photos and FT-IR curves of Part B0 and Part B15.

The chemical reaction mechanism between DMMP and ethylene glycol, used to generate PPCP, is shown in Scheme 1. In order to demonstrate the successful synthesis of PPCP and its chemical bonding reaction with PAPI, FT-IR curves of raw materials, PPCP, Part B0, and Part B15 are shown in Figure 1.

As shown in Figure 1a, owing to the formation of hydrogen bonds between the hydroxyl groups and P=O groups in PPCP, the stretching vibration absoption peak of P=O in PPCP was located at 1244 cm^−1^, while the redshift value compared with DMMP was 34 cm^−1^. Characteristic absorption peaks at 903 cm^−1^ and 724 cm^−1^ corresponded to the stretching vibration of P-O-C and P-C bonds, respectively [41]. Meanwhile, the stretching vibration absorption peak of O-H can be obviously seen at 3390 cm^−1^ in the curve of PPCP. These results preliminarily indicate that PPCP is successfully obtained.

Figure 1b shows the FT-IR curves of PAPI (Part B0) and Part B15. Compared with Part B0, a new peak appeared at 1744 cm^−1^ in Part B15, which corresponds to the symmetrical stretching vibration absorption peak of the C=O bond in the carbamate group (-O-CO-NH-) structure owing to the reaction between PPCP and PAPI, as shown in Scheme 3. In this study, a characteristic absorption peak at 1520 cm^−1^, assigned to the benzene ring, also represents an internal standard peak [8]. Compared with Part B0, the relative intensity of the isocyanate group’s absorption peak at 2278 cm^−1^ in Part B15 is weakened. These results effectively indicate that PPCP can form chemical bonds with PAPI. Moreover, compared with the clarified Part B0, a digital photo shows that the state of Part B15 is turbid, further demonstrating the reaction between PPCP and PAPI.

### 3.2. Cell Morphology of Partial RPIF

**Figure 2:**
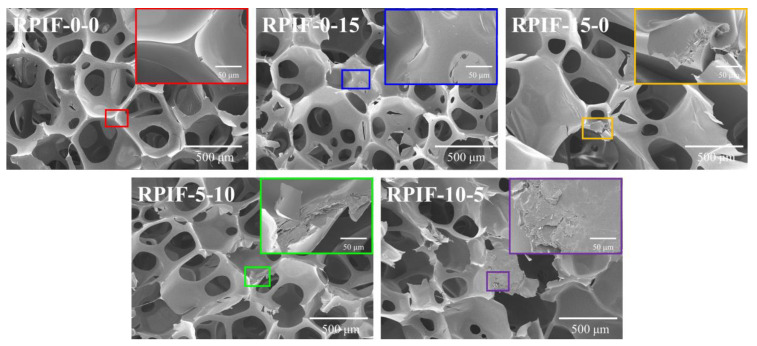
SEM images of partial RPIF.

SEM images of partial RPIF are shown in Figure 2. The density of all RPIFs is within the range of 28~33 kg/m^3^, and is listed in Table S2. Figure 2 shows that all RPIFs present a nearly spherical bubble structure, and the cellular windows are smooth. The cell structure of RPIF-0-15 is very similar to that of RPIF-0-0, while there is no other substance in the cellular skeletons. Meanwhile, the significant advantage, as derived from a previous study in which only common phosphorus liquid flame retardants were applied, is that the cellular structure of RPIF-0-15 is more regular [11]. However, when EG is used, as shown in SEM images of RPIF, including RPIF-15-0, RPIF-5-10, and RPIF-10-5, layer structures of EG can be clearly observed in the walls and skeletons of partial bubbles. This reveals the form in which EG exists in RPIF cells.

For the effect of PPCP and EG on the cell size of RPIF, the average cell sizes of all RPIFs are calculated and listed in Table S2 of the supporting information. The average cell size is within the range of 0.49~0.61 mm. Although the change in cell size is not very obvious, some rules can also be inferred from the analysis. It is found that the introduction of PPCP gradually decreases the cell size of RPIF, which may be caused by the increased viscosity of Part B owing to the reaction between PPCP and PAPI, which will restrain the expansion of cells and decrease their size. For EG, the average cell size of RPIF presents a trend of first decreasing and then increasing with the increase in EG dosage. This change may be due to the destruction of nano-filler for the stability and surface tension of foaming slurry, which then leads to the crosslinking speed lagging behind the foaming speed during the foaming process; some gas escapes from the cells, so the cells are not fully expanded and their size is decreased. When the EG dosage is continuously enhanced, the viscosity is gradually enhanced, and this enhances the strength of foaming intermediately; then, the escape of gas is restrained, and cells are effectively expanded. The average cell size is finally enhanced.

### 3.3. Element Content and Chemical Structure of RPIF Matrix Resin

**Figure 3:**
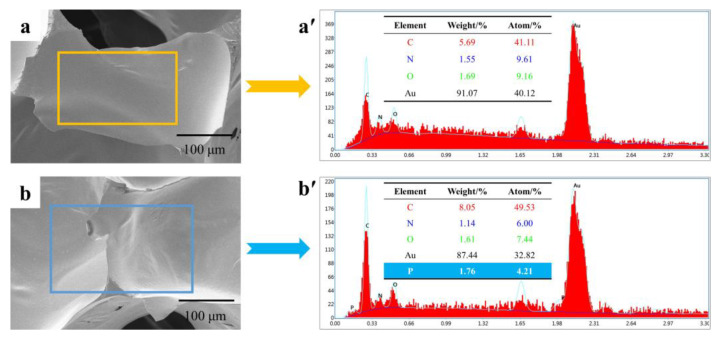
EDS results of RPIF-0-0 (**a**,**a′**) and RPIF-0-10 (**b**,**b′**).

**Figure 4:**
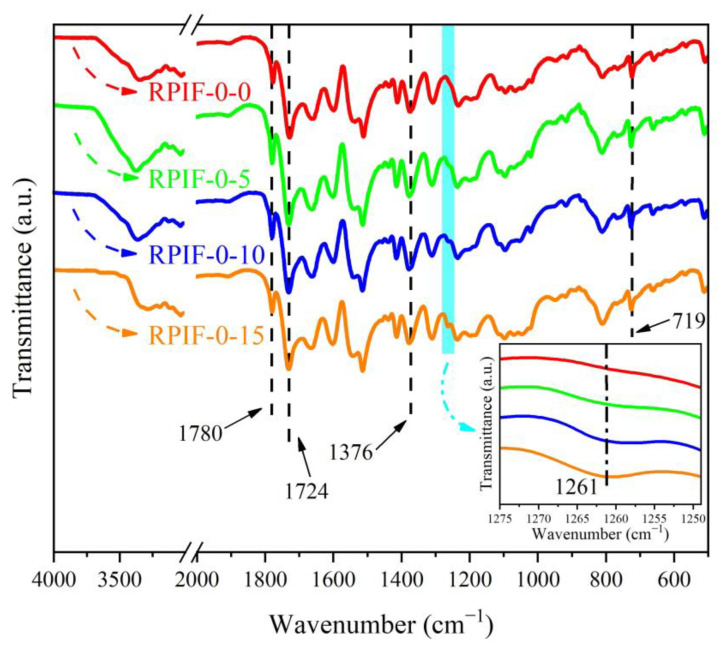
FT-IR curves of RPIF-0-P series.

EDS data of RIF-0-0 and RIF-0-10 are presented in Figure 3. The appearance of the element P in Figure 3b′ effectively demonstrates the existence of P in RIF-0-10. To further prove the chemical bonding results of PPCP in the RPIF matrix resin, FT-IR curves of the RPIF-0-P series are created, as shown in Figure 4. Characteristic absorption peaks of polyimide at 1780 cm^−1^, 1724 cm^−1^, 1376 cm^−1^, and 719 cm^−1^ are clearly observed in all curves. In addition, at 1261 cm^−1^, the weak peak gradually increased in intensity with the increasing PPCP dosage. This corresponds to the stretching vibration absorption peak of P=O in the PPCP structure. This further demonstrates the grafting of phosphorous flame-retardant chain segments in the RPIF resin. Therefore, the results mentioned in 3.1 and this section comprehensively prove the successful chemical introduction of phosphorous flame-retardant chain segment into RPIF resin.

### 3.4. LOI of RPIF

**Figure 5:**
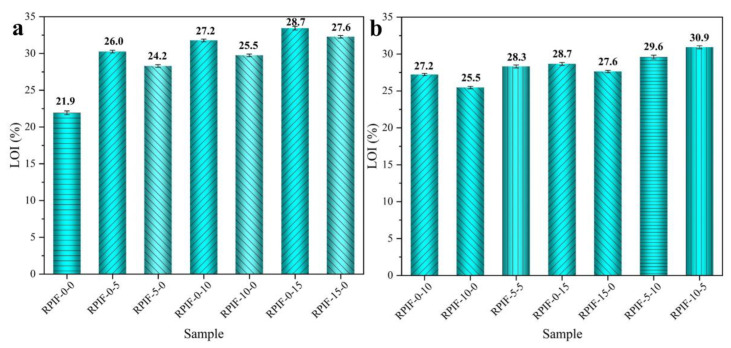
Difference and comparison of effects of PPCP and EG on LOI of RPIF, PPCP or EG alone used (**a**), and combination use of PPCP with EG (**b**).

LOI, actual combustion behavior, and CCT data can be applied to evaluate the flame retardancy of RPIF. A comparison of the effects of PPCP and EG alone on the LOI of RPIF is presented in Figure 5a. It shows that the increased dosage of PPCP and EG can gradually enhance the LOI of RPIF. The LOI of RPIF-0-0 is only 21.9%, but those of RPIF-0-15 and RPIF-15-0 reach 28.7% and 27.6%, respectively. It was clearly shown that RPIF prepared by only introducing PPCP possesses a higher LOI than that obtained by only adding EG. In this study, RPIF-0-5 and RPIF-5-0 present the greatest difference, while the LOI value of RPIF-0-5 is 1.8% higher than that of RPIF-5-0, corresponding to a relative enhancement of 7.4%. These results prove that PPCP shows better flame-retardant efficiency than EG for RPIF. Meanwhile, it indicates that RPIF will become more difficult to ignite when PPCP is introduced, meaning more oxygen and a higher oxygen concentration are needed. It benefits from the earlier decomposition of the PPCP component than the matrix resin, which is shown in Section 3.5. This leads to the large number of PO radicals filling in the surrounding gas environment; the post-generated H radical and hydrocarbyl radical from the decomposition of the matrix resin will be quickly quenched by PO radicals, and their concentration also decreases. Thus, if the combustion reaction between active free radicals and oxygen is to be triggered, the oxygen concentration must be enhanced, corresponding to an improvement in LOI.

Figure 5b reflects that the combination of PPCP and EG synergistically enhances the flame retardancy of RPIF. Taking RPIF-5-5, RPIF-10-0, and RPIF-0-10 as examples, all possess a sum of EG and PPCP at 10 wt. %, but the LOI of RPIF-5-5 at 28.3% distinctly surpasses those of RPIF-0-10 (at 27.2%) and RPIF-10-0 at (25.5%). Moreover, RPIF-5-10 and RPIF-10-5 all present higher LOIs than RPIF-15-0 and RPIF-0-15. These promising results preliminarily indicate that, when the distribution ratio of EG to PPCP is set in the range of 1:2 to 2:1, they exhibit significant synergy in relation to the flame retardancy of RPIF. In addition, compared with RPIF-5-10, the LOI value of RPIF-10-5 changes by 4.4%, which reflects that a higher dosage ratio of EG to PPCP leads to a higher LOI value of RPIF in this system.

When EG is used alone, long cracks are easily formed on the surface of the char layers owing to the internal stress caused by EG expansion during the heating or combustion process. This greatly reduces the flame-retardant efficiency of EG [42,43]. Thus, PPCP shows a better flame-retardant efficiency than EG when they are at same dosage. However, when EG and PPCP are present in RPIF at the same time, the crosslinked phosphorus oxide and phosphorus carbon compounds formed by PPCP pyrolysis during heating or combustion can effectively bond the worm-like loose porous char residues formed by EG. They can then prevent the formation of long cracks and form a dense carbon layer to enhance the fire resistance of the RPIF matrix [33]. Therefore, the combination of PPCP and EG can synergistically improve the flame retardancy of RPIF.

### 3.5. Thermal Stability of RPIF

**Figure 6:**
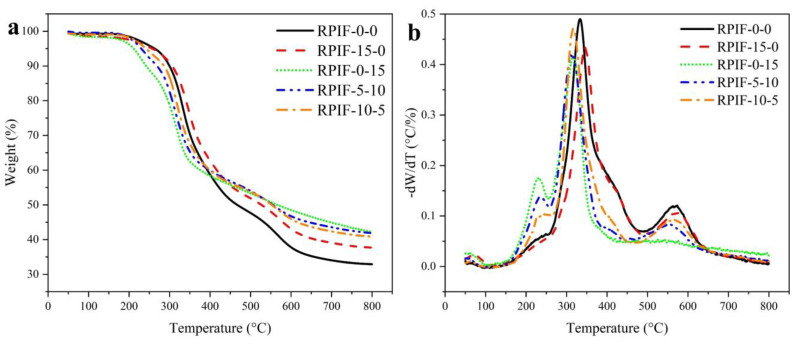
TGA (**a**) and DTG (**b**) curves of RPIF.

TGA and derivative thermogravimetry (DTG) are used to evaluate the thermal stability of RPIF. The TGA and DTG curves of RPIF-0-0 and RPIF possessing a total dosage of EG and PPCP at 15 wt. % are shown in Figure 6. The decomposition temperature at 5% weight loss (T_5%_) and residual weight at 800 °C (R_w_) are listed in Table S1. RPIF presents three decomposition regions in the ranges of 160~257 °C, 257~475 °C, and 475~640 °C, respectively [8].

For RPIF-0-0, the small region from 160 to 257 °C refers to the further evaporation of residual and generated small molecules, and the T_5%_ reaches 263 °C. However, the introduction of PPCP significantly improves weight loss in this region. The T_5%_ of RPIF-0-15, which has the highest dosage of PPCP, is directly reduced to 211 °C. The second region corresponds to the decomposition of polyurea. From the DTG curves, it is obvious that the temperature at the maximum decomposition rate of the second region drops after PPCP is introduced, whereas the use of EG can push it to a higher value. The last region of 450~640 °C ascribes to the decomposition of PI. Moreover, a higher PPCP dosage leads to the slower decomposition of PI, and all RPIFs, including samples to which only PPCP is applied, show higher R_w_ than RPIF-0-0.

The above data indicate that the PPCP incorporated in RPIF can be pyrolyzed in the first region, and also releases PO radicals as previous reported, which leads to the increase in T_5%_ [28]. To a certain extent, the introduction of PPCP reduces the thermal stability of RPIF. In addition, the produced phosphoric acid derivatives coming from the PPCP segments’ decomposition effectively promotes the degree of carbonization of polyurea and PI in the following decomposition region, as indicated by the increased R_w_ value. These actions can facilitate the formation of thicker and denser char layers. The generated char layers act as fireproof layers to inhibit contact between the fire and the internal matrix, playing an important part in the high flame retardancy of RPIF.

### 3.6. Combustion Behavior

**Figure 7:**
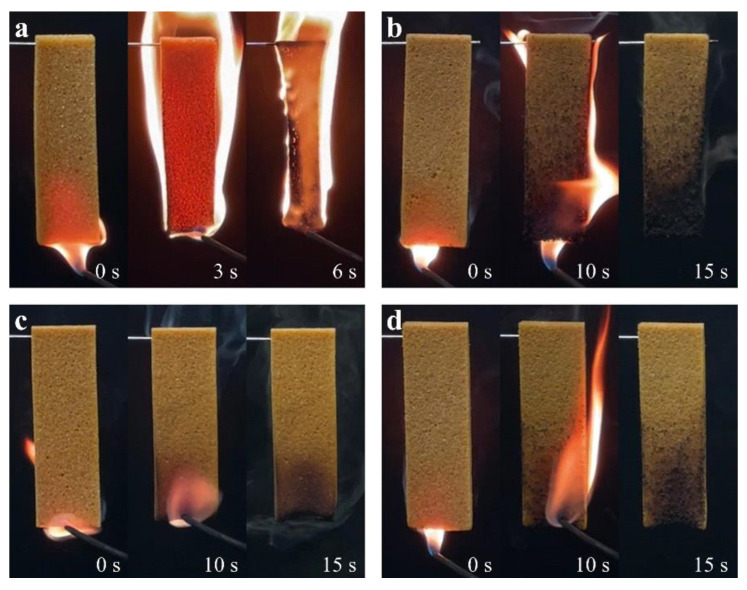
Digital photos of partial RPIF ignited at different times, (**a**) RPIF-0-0, (**b**) RPIF-5-0, (**c**) RPIF-0-5, and (**d**) RPIF-5-5.

The actual ignition test is used to intuitively characterize the effects of PPCP and EG on the flame retardancy of RPIF. The photos are presented in Figure 7. They show that RPIF-0-0 can be quickly ignited in less 3 s and entirely burned out before removing the igniter. However, no dripping and a better shape-retention ability are found when comparing with RPUF. It demonstrates that common RPIF achieves better use safety than RPUF. In addition, although RPIF-5-0, RPIF-0-5, and RPIF-5-5 are combusted for more than 10 s, they all can achieve self-extinguishing after removing the igniter, and still retain their original size. RPIF-0-5 and RPIF-5-5 are basically undamaged. These results indicate the positive effects of EG and PPCP on enhancing the flame retardancy and use safety of RPIF.

The CCT results can provide information close to the practical situation for flammability and smoke release performance [8,33]. The flammability parameters, including PHRR and THR at 155 s, are listed in Table S2. Figure S1 shows the heat release rate (HRR) and THR curves of RPIF with EG or PPCP introduced alone. The results show that EG and PPCP effectively reduce the HRR and PHRR of RPIF. So, the flame retardancy is effectively enhanced. However, EG is more effective in weakening the violent combustion behavior of RPIF than PPCP. It can thus better enhance the safety of RPIF when applied alone. A detailed analysis and comparison are presented in the supporting information section.

**Figure 8:**
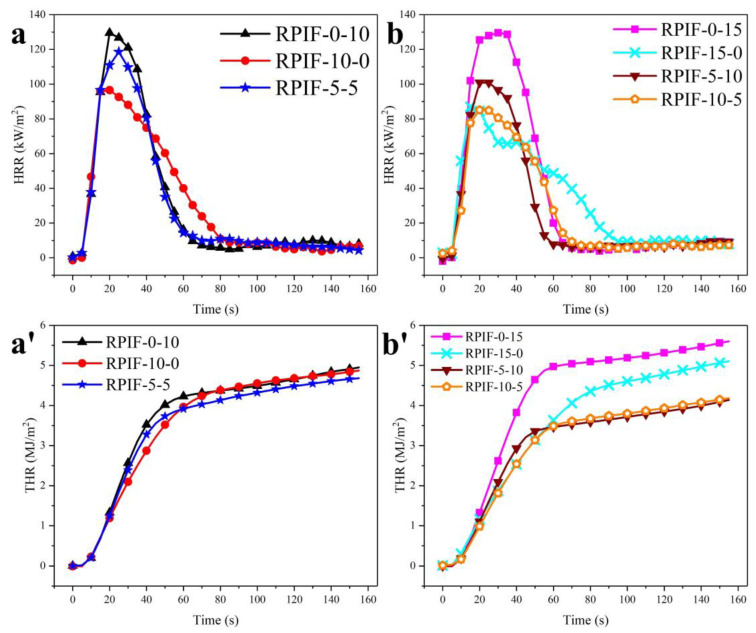
HRR and THR curves of RPIF with total dosages of EG and PPCP at 10 wt. % (**a**,**a′**) and 15 wt. % (**b**,**b′**).

To study the synergistic effects of EG and PPCP on the flame retardancy, combustion behavior, and safety of RPIF, two series of RPIF have been selected. The first includes RPIF-0-10, RPIF-10-0, and RPIF-5-5, each with a total dosage of EG and PPCP of 10 wt. %. The second comprises RPIF-0-15, RPIF-15-0, RPIF-5-10, and RPIF-10-5, each with a total dosage of EG and PPCP of 15 wt. %. Figure 8 presents HRR and THR’s results. In the first series, the PHRR of RPIF-5-5 is lower than that of RPIF-0-10, but it is still higher than that of RPIF-10-0. However, the THR at 155 s of RPIF-5-5 is only 4.68 MJ/m^2^, and the THR curve becomes lower than that of RPIF-0-10 and RPIF-10-0 from about 60 s. For the second series, interestingly, the PHRR and THR of RPIF-10-5 are all lower than those of RPIF-15-0 and RPIF-0-15, while THR at 155 s is only 4.18 MJ/m^2^. Furthermore, from the beginning, the THR curve of RPIF-10-5 is consistently lower than those of the other RPIFs in this series.

These results effectively demonstrate that the combined use of EG and PPCP can further improve the flame retardancy of RPIF. Furthermore, when comparing the PHRR and THR of RPIF-10-5 and RPIF-5-10, EG exhibits greater synergistic effects of inhibiting the heat releasing behavior of the RPIF combustion flame than PPCP. This, combined with the LOI results obtained in Figure 5, show that, when the distribution ratio of EG to PPCP is set within the range of 1:2 to 2:1, they exhibit significant synergy for the flame retardancy of RPIF. It can be further stated that the most suitable positive synergy ratio of EG and PPCP for the RPIF system is about 2:1. On the other hand, the price of commercialized EG is lower than synthesized PPCP. Therefore, the combined introduction of EG with PPCP to RPIF can not only decrease the dosage of PPCP, but also further enhance the flame retardancy of RPIF.

From the comprehensive analysis of the LOI, HRR, and THR results, EG can be inferred to be an ideal synergistic flame retardant for PPCP in the RPIF system, while the combined use of EG and PPCP leads to better flame retardancy effects for RPIF than singular use. The source of positive synergy is the formation of a dense flame and a heat barrier carbon layer. During the combustion process, the worm-like graphite carbon formed by EG can provide a primitive unit of a large carbon source for the formation of a carbon layer. The decomposition of RPIF offers a carbon network skeleton to ensure the distribution of worm-like graphite carbon in the 3D interspace. Finally, the crosslinked phosphorus oxide and phosphorus carbon compounds formed by PPCP pyrolysis can effectively bond the worm-like loose porous char and carbon network skeleton, or themselves. The effective linkages prevent the formation of long cracks, so a dense and more stable carbon layer is fabricated to realize enhanced fire resistance and use safety of RPIF. Moreover, as an increasing amount of EG is used, a larger carbon source is supplied, which is more conducive to the formation of a carbon layer, so the most suitable positive synergy ratio of EG and PPCP for RPIF system is about 2:1 in this study.

### 3.7. Smoke Release and Toxic Fume Generation

Thick smoke particles and their toxic components are major factors causing casualties in fire [33]. Smoke production release (SPR), peak of SPR (PSPR), carbon monoxide production (COP), total carbon monoxide production (TCOP) obtained by CCT, Ds, and HCN concentration in smoke are analyzed. The results of PSPR, TSP at 155 s, and Ds max of partial RPIF are listed in Table S2.

The SPR and TSP curves of RPIF with only PPCP or EG introduced are shown in Figure S2. The results clearly demonstrate the negative effect of PPCP on the smoke release performance of RPIF, and PSPR and TSP all show dramatic increases. The smoke toxicity and hazard to human health of RPIF during the combustion process are also enhanced, attributable to the gas-phase flame retardancy process and mechanism, as stated in the supporting information. However, EG exhibits diametrically opposite effects to PPCP regarding the smoke release behavior of RPIF. EG shows an effective smoke-suppressant effect on RPIF owing to the adsorption of particulate matter generated during RPIF combustion by porous and worm-like graphitic carbon. Moreover, this research also finds that EG presents more excellent smoke suppression behavior than flame retardancy performance for RPIF. A detailed analysis and comparison are presented in the supporting information.

**Figure 9:**
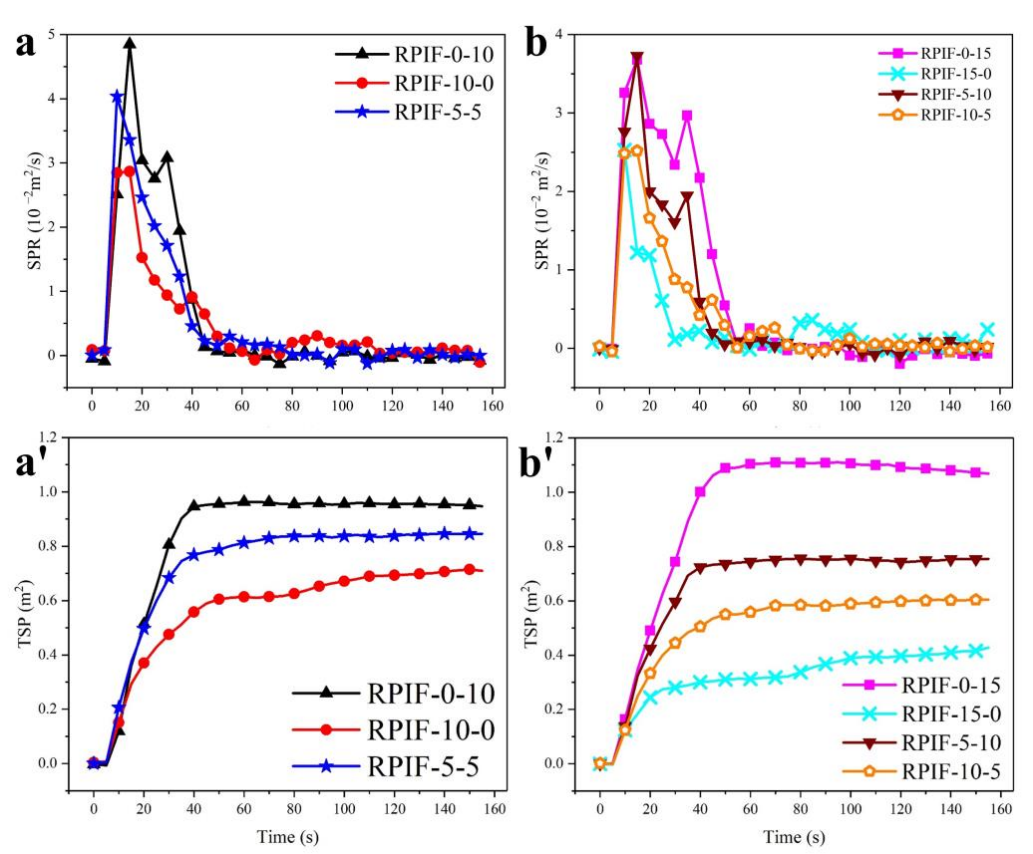
SPR and TSP curves of RPIF with the total dosage of EG and PPCP at 10 wt. % (**a**,**a′**) and 15 wt. % (**b**,**b′**).

Figure 9 shows the actual smoke suppression effect of simultaneously employing EG and PPCP on RPIF. For RPIF with the sum of EG and PPCP at 10 wt. % (Figure 9a,a′), the PSPR and TSP of RPIF-5-5 are 0.040 m^2^/s and 0.84 m^2^, respectively. These results are between those of RPIF-10-0 and RPIF-0-10. For RPIF with the sum of EG and PPCP at 15 wt. % (Figure 9b,b′), the PSPR and TSP of RPIF-10-5 and RPIF-5-10 are also between those of RPIF-0-15 and RPIF-15-0. These results are logical given the exact opposite effects of EG and PPCP on the smoke release behavior of RPIF.

Fortunately, it is determined that the PSPR and TSP of RPIF-10-5 are all even lower than those of RPIF-10-0. The additional introduction of 5 wt. % PPCP is supposed to increase PSPR and TSP according to the above-mentioned results and those in the supporting information. These promising results effectively show that the combination of EG and PPCP can synergistically reduce smoke release. Coincidentally, the PSPR and TSP of RPIF-5-10 are all even lower than those of RPIF-5-5. The synergistic effect of EG and PPCP on the smoke release behavior of RPIF is fully manifested. Owing to this synergistic effect, the incorporation or increased dosage of an additive, which should cause enhanced smoke generation, unexpectedly brings about a decrease in smoke release. The key factor is the strengthened char layer for pyrolysis and the bonding effect of the PPCP chain. The phosphoric acid derivatives generated from the pyrolysis of PPCP can adhere to the worm-like loose porous char layer formed by EG. Therefore, the loose char layer cannot be stripped out or blown out of the condensed phase. Furthermore, the greater the amount of PPCP that is incorporated, the stronger the reinforcement that will be produced, and more bonding between the worm-like loose porous char and carbon network skeleton will occur. Thus, a denser and more porous char layer is formed. Compared with the unanchored worm-like loose porous expanded EG, this condensed and fixed porous structure formed by EG and the crosslinked structure can achieve the greater absorption of smoke. Meanwhile, the condenser and condenser char layers block the path of smoke release and delay such release, which directly increases the amount of time for which smoke exists in the carbon layer. This in turn helps the expanded EG to absorb all of the smoke. Thus, an increasing amount of smoke is absorbed and filtered. Hence, although the increased PPCP dosage leads to an increase in TSP in the interior of RPIF during the combustion process, the synergistic effects of EG and PPCP can effectively decrease the TSP value due in the unique carbon layer structure. On the other hand, the formation of a dense and more stable char layer also effectively acts as a fire barrier, which restricts the spread of flame and heat to RPIF’s interior, so the combustion of resin can also be lowered.

In summary, for RPIF-10-0 and RPIF-5-5, the continuous increase in PPCP dosage effectively enhances the flame retardancy and use safety of RPIF. These results further demonstrate the reasonableness and correctness of using EG and PPCP combination in the RPIF system. These two additives play complementary and positive synergistic roles to enhance the safety of RPIF. They are an ideal match.

**Figure 10:**
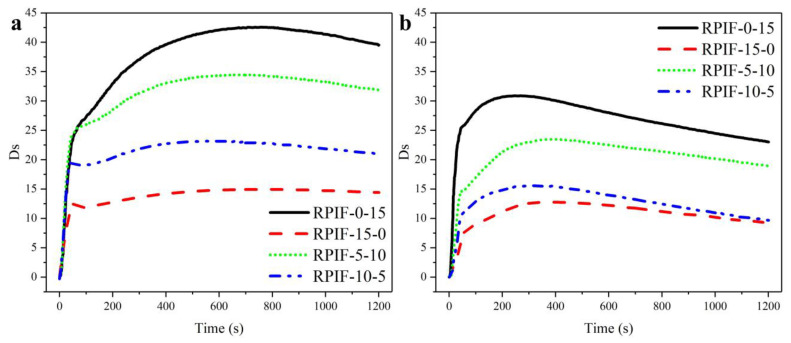
Ds curves of RPIF with total dosage of EG and PPCP at 15 wt. %: (**a**) test without flame condition, (**b**) test with flame condition.

Ds is an important parameter used to evaluate the safety and smoke hazards of a polymer. There are clear requirements for the Ds value of materials in fire test procedures (FTP) to ensure their qualified security. So, Ds evaluation is important for polymeric foams. Figure 10 shows Ds curves of RPIF with the total dosage of EG and PPCP of 15 wt. % without or with a flame. The maximum value of the Ds curve during whole test process over 1200 s is defined as Ds max. The Ds max values are listed in Table S2. In this series of samples, under two different characterization conditions, all the results show that a higher proportion of added EG leads to a lower Ds curve and Ds max of RPIF. The Ds max values of RPIF-10-5 under different test conditions are all lower by about 33% than RPIF-5-10.

The Ds results are consistent with the regularity of TSP data in CCT. This further supports the importance of the combined use of EG and TCPP for ensuring use safety of RPIF. Meanwhile, the Ds max in the presence of a flame is lower than that without a flame. This is because the flame condition can promote the combustion degree and velocity. This is also in line with the classic combustion behavior. Moreover, the Ds max of all samples is lower than 45, which means that the safety of RPIF modified by EG and PPCP in this study meets the requirements of FTP rules for thermal insulation and sound absorption foamed materials. These results prove again that the introduced PPCP increases the generation of smoke particles, but the composited EG can effectively reduce the releasing of smoke particles and weaken the adverse effects on smoke release behavior caused by PPCP.

Meanwhile, in Table S2 and Figure 5, although the corresponding CCT results, including the PHRR, THR, PSPR, and TSP of RPIF-10-5 and RPIF-5-10, are all lower than that of RPIF-0-0, and the LOI are all higher than that of RPIF-0-0, only the Ds max values of RPIF-10-5 under different test conditions are still all lower than that of RPIF-0-0, while those of RPIF-5-10 under different test conditions are all higher than for RPIF-0-0. Thus, the comprehensive evaluations of the smoke release behavior and fire behavior tests all preliminarily show that RPIF-10-5 possesses the optimal use safety in this study, and the ratio of EG to PPCP of 2:1 is a suitable value for the RPIF system.

**Figure 11:**
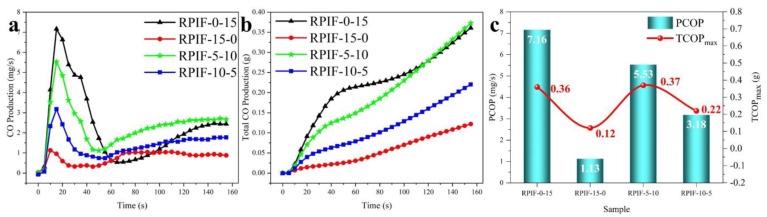
COP (**a**) TCOP (**b**) curves, PCOP and TCOP_max_ (**c**) of RPIF with total dosages of EG and PPCP at 15 wt. %.

The generation of CO is a very important factor to assess smoke toxicity, toxic fume generation, and the use safety of a polymer during the burning process [44,45]. Figure 11 shows the peak value of the CO production curve (PCOP), as well as the COP and TCOP curves of RPIF with total dosage of EG and PPCP at 15 wt. %. Curves with the same colors and symbols as in Figure 11a,b correspond to the same sample. It can be clearly observed that a higher EG proportion corresponds to lower PCOP and TCOP values under the same total dosage. These data indicate that EG can effectively inhibit the release of CO, while the introduction of PPCP directly promotes CO generation. It also demonstrates the opposite effect of PPCP and EG on toxic fume generation behavior. The PCOP and TCOP of RPIF-10-5 are 3.18 mg/s and 0.22 g, respectively, which also indicate an obvious reduction compared to RPIF-5-10. These results are consistent with those of smoke release behavior, as above-mentioned; they also match with the results obtained in Section 3.6, which state that the most suitable positive synergy ratio of EG and PPCP for the RPIF system is about 2:1 in terms of flame retardancy performance. It further means that the increased proportion of EG in the composited additive can effectively enhance the flame retardancy, use safety, and low toxic smoke index. This result also indicates that although PPCP is bonded with matrix resin, its existence remains a hugely unfavorable factor for the controlling of the generation and releasing of toxic fumes and smoke. By extinguishing the flame and capturing the oxygen-containing free radicals of PO generated and released from the phosphorous flame-retardant chain segment, this effect induces the incomplete combustion of resin, and finally leads to an increase in CO generation [30]. However, the common use of EG can effectively limit these drawbacks of PPCP to ensure the fire prevention behavior of RPIF. Thus, EG can be selected as an ideal partner for PPCP in terms of enhancing the use safety of RPIF.

The weakened effect of EG on COP, PCOP, and TCOP is also due to the following reasons, as previous stated. During the combustion process, EG can release steam and other non-flammable gases, so the concentration of gas in the combustion zone is effectively reduced [38]. Most important is the fact that the generated porous structure of EG also plays an important role in the absorption of toxic fume.

**Figure 12:**
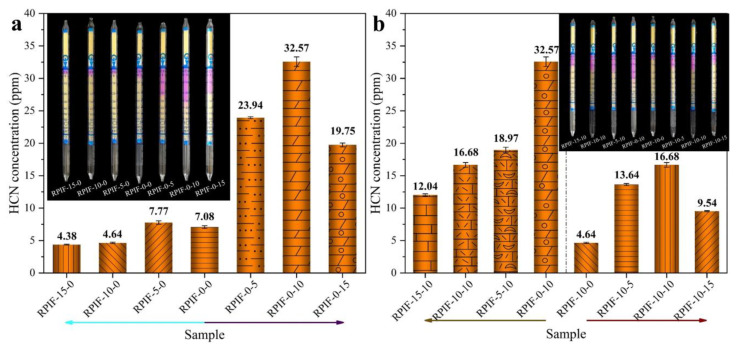
Difference and comparison of the effects of PPCP and EG on the HCN concentration of the RPIF combustion process, PPCP or EG alone used (**a**), and combination use of PPCP with EG (**b**).

HCN has been considered as the most toxic gas in the smoke produced by polymer combustion [46]. The HCN concentration in RPIF combustion flue gas is portrayed in Figure 12. Figure 12a presents the HCN concentrations of the RPIF-E-0 and RPIF-0-P series. The HCN concentrations of RPIF in the RPIF-E-0 series are all within a low range and at a relatively safe level. For RPIF-10-0 and RPIF-15-0, the HCN concentrations are only 4.64 ppm and 4.38 ppm, respectively. They correspond to reductions of 34% and 38% compared with RPIF-0-0, respectively. This is also due to the distinctive adsorption effect of the loose and porous expanded EG, and the enhanced flame retardancy of RPIF. However, for the RPIF with PPCP, the HCN concentrations are all higher than that of RPIF-0-0 in this study. For the RPIF-0-P series and RPIF-10-P (EG dosage fixed at 10 wt. %, PPCP dosage ranges from 0 to 15 wt. %) series, regardless of whether EG is commonly used, the HCN concentrations all present a significant increasing trend with increasing PPCP dosage. The falling mutation does not appear until the PPCP dosage reaches 15 wt. %. This change can be clearly proven by reference to the columns in Figure 12. The value of RIF-0-10 even reaches 32.75 ppm, corresponding to an increase of 360% compared with RPIF-0-0. That is deemed a highly toxic state. That of RIF-10-10 also reaches 16.68 ppm and corresponds to an increase of 259% compared to RPIF-10-0. However, the continuous increase in PPCP dosage leads to a decrease in HCN concentration, and the values of RPIF-0-15 and RPIF-10-15 decline to 19.75 ppm and 9.54 ppm, respectively. The HCN concentration of RPIF-10-15 is only 2.46 ppm higher than that of RPIF-0-0, and they are basically at same toxicity level. However, the HCN concentrations of all RPIF applied with PPCP in this study are higher than that of RPIF-0-0, which reflects the negative effect of PPCP on the toxic fume generation of RPIF.

It has been suggested that structures containing an amide structure (-COR-NH-) are broken down to form HCN [47,48]. In this study, the introduced PPCP reacts with isocyanate to form urethane, which contains an amide structure; the increase in PPCP dosage directly and continuously enhances the contents of amide group in RPIF, so the HCN concentration will be dramatically increased. The decrease in HCN concentration after the PPCP dosage reaches 15 wt. % may be due to the high flame retardancy of RPIF-0-15 and RPIF-10-15. Because the test conditions in the NES-713 smoke toxicity test chamber correspond to a typical atmospheric environment, the oxygen concentration is also about 21%, so when the flame retardancy of RPIF reaches a certain level, it is difficult to ignite and decompose under normal flame conditions. Then, the original matrix resin and size will be maintained as in Figure 7, so the HCN concentration is reduced.

Furthermore, from the HCN results of the RPIF-E-10 series (PPCP dosage is fixed at 10 wt. %, EG dosage ranges from 0 to 15 wt. %) compared with the RPIF-10-P and RPIF-0-P series, it is clearly shown that EG plays an effective and ideal partnering role with PPCP in reducing HCN concentration. For the RPIF-E-10 series, the combined use of only 5 wt. % EG can directly reduce the HCN concentration by about 42%, and the efficiency of the reduction reaches 2.72 ppm per 1 wt. % EG. When the EG dosage is continuously enhanced, the HCN concentration is further declined, and when that of RPIF-15-10 is only 12.04 ppm, the weakening efficiency of EG for HCN is still as high as 1.37 ppm per 1 wt. % EG. These all owe to the effective absorption action of the porous structure in expanded EG in relation to the generated HCN. So, the smoke toxicity is effectively ameliorated.

Compared with RPIF-10-P and RPIF-0-P, the fixed 10 wt. % EG dosage lowers the HCN concentration by 10.30 ppm, 15.89 ppm, and 10.21 ppm for RPIF-0-5, RPIF-0-10, and RPIF-0-15, respectively. Further calculations show that the combined use of only 10 wt. % EG can directly decrease the HCN production level to about half of all the RPIF in the original RPIF-0-P series, even though the PPCP dosage is gradually enhanced. Such a large reduction in HCN concentration illustrates the effective action of EG in decreasing the toxic gas production caused by the introduction of PPCP. So, the simultaneous incorporation and combination of EG with PPCP can effectively inhibit the serious toxic gas releasing problem caused by PPCP. This owes to the enhanced flame retardancy of RPIF, which can decrease the decomposition of the matrix resin when subjected to fire, as well as the effective absorption of toxic fumes by expanded EG.

According to the release results of toxic gas, including the CO and HCN studied in this paper, it can be inferred that EG is an excellent and ideal partner for PPCP when preparing RPIF with excellent use safety in the low-toxic gas generation field during the combustion process.

### 3.8. Discussions of the Synergistic Flame-Retardant Mechanism between EG and PPCP

**Figure 13:**
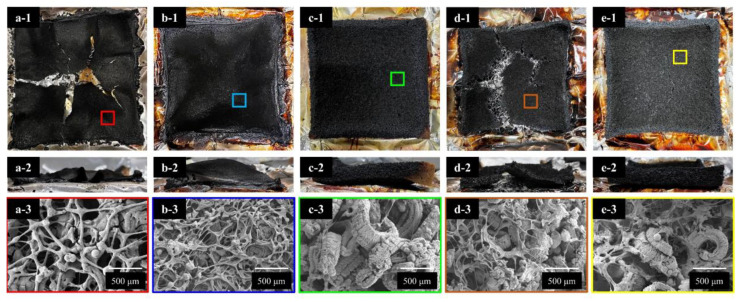
Digital photographs and SEM images of char residues of RPIF-0-0 (**a-1**)–(**a-3**), RPIF-0-15 (**b-1**)–(**b-3**), RPIF-15-0 (**c-1**)–(**c-3**), RPIF-5-10 (**d-1**)–(**d-3**), and RPIF-10-5 (**e-1**)–(**e-3**) after CCT.

As above-mentioned, under the synergistic effects of EG and PPCP, RPIF shows excellent use safety. In this section, the flame-retardant effect, smoke-suppressant effect, and toxic fume reduction mechanisms are further proven and discussed according to the composition and morphology of char residues left after CCT. Digital camera and SEM images of partial RPIF (total dosage of EG and PPCP at 15 wt. %) after CCT are shown in Figure 13. It is obvious that the residue of RPIF-0-0 is thin and has wide cracks (Figure 13(a-1),(a-2)). Meanwhile, there are many connected pores and holes in the interior of the residue (Figure 13(a-3)), which allow the free passage of gas and smoke. No cracks are observed on the surface of the RPIF-0-15 residues (Figure 13(b-1)), while the numbers of connected pores and holes in the interior also decrease significantly (Figure 13(b-3)). A comparison of Figure 13(a-2),(b-2) indicates that PPCP had little effect on residue thickness. The surface of the RPIF-15-0 residue (Figure 13(c-1)) also barely has any cracks. Compared with RPIF-0-15 and RPIF-0-0, the char layer thickness of RPIF-15-0 residue (Figure 13(c-2)) is enhanced owing to the expansion of EG. However, Figure 13(c-3) shows that almost no linked skeleton structure can be found after combustion, and the expanded EG is only in a worm-like graphite state and loosely distributed. Although this worm-like loose porous char layer has an excellent barrier effect against a flame, the loosened state also leads to the slow passage of oxygen and heat, and RPIF-15-0 exhibits secondary combustion in the CCT and double peaks in the HRR curve under these two effects. However, when EG and PPCP are used in combination, the linked and dense char layer network (Figure 13(d-3),(e-3)) composed of a residue skeleton and worm-like graphite can be observed in the microstructure of the char layer, owing to the bonding between the worm-like loose porous char and carbon network skeleton. The phosphoric acid derivatives formed by the thermal decomposition of the phosphorous flame-retardant chain segment in PPCP are deposited layer by layer, and crosslink with the porous expanded EG, so the compactness of the char layer is enhanced and the number of pores is reduced. These changes in structure can have great flame-retardant and smoke-suppressant effects. Moreover, the thickness of char layer is enhanced, so the isolation effect for flames is also improved. Thus, the combined use of EG and PPCP is an effective way to greatly enhance the use safety of RPIF.

**Figure 14:**
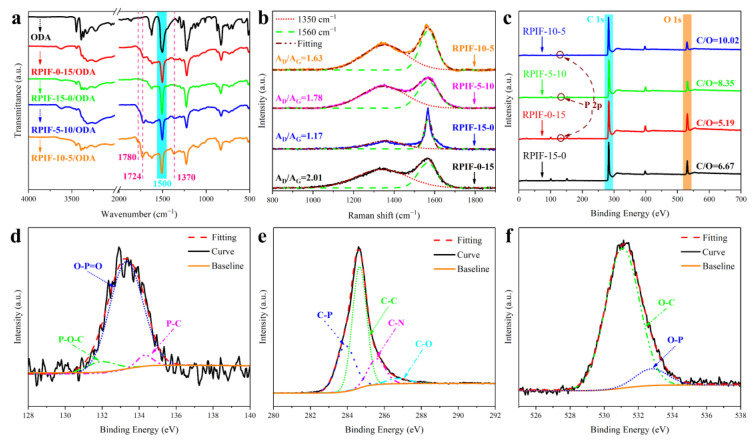
FT-IR spectra (**a**), Raman spectra (**b**), and XPS survey spectra (**c**) of the residue of RPIF with a total dosage of EG and PPCP at 15 wt. % after CCT; high-resolution P 2p (**d**), C 1s (**e**), and O 1s (**f**) XPS spectra of RPIF-10-5 residue after CCT.

Because no characteristic absorption peak in the FT-IR curves of RPIF residues can be used as the internal standard peak, in order to analyze the composition of char residue and burning degree, the residue is mixed with 4,4’-oxydianiline (ODA) at a mass ratio of 4:1, and the FT-IR curves of the corresponding mixtures are shown in Figure 14a. A characteristic absorption peak at 1500 cm^−1^ owing to the benzene ring in ODA is taken as the internal standard peak [8]. The results show that the relative intensities of PI characteristic absorption peaks at 1780 cm^−1^, 1724 cm^−1^, and 1370 cm^−1^ in the RPIF-10-5 residue are obviously higher than those of other RPIF residues. This indicates that the char layer of the RPIF-10-5 residue has outstanding heat and oxygen insulation properties, and the matrix burns incompletely at a heat flux of 35 kW/m^2^ [8]. Thus, RPIF-10-5 shows low CCT results and high flame retardancy.

The Raman spectra of residues all show strong characteristic peaks at 1350 cm^−1^ and 1560 cm^−1^; they belong to D and G bands, respectively (Figure 14b). The lower the peak area ratio of the D and G bands (AD/AG), the higher the graphitization degree of the char layer [41]. It is obviously observed that the specific value of the RPIF-15-0 residue is lower than that of the other RPIFs. Hence, the graphitization degree of RPIF-15-0 is the highest. This is caused by the worm-like graphite in the EG. Generally speaking, a higher graphitization degree corresponds to a higher fire-resistant behavior of the char layer; this result also demonstrates the enhanced flame retardancy of RPIF with increased EG dosage. However, SEM images of the RPIF-15-0 residue show that the residue formed by EG is loose and porous. This state can be easily altered upon disturbance by a flame, which reduces the flame-retardant effect. However, RPIF-10-5 residue not only has a higher degree of graphitization (AD/AG = 1.63) than RPIF-0-15 residue, but it also possesses a denser char layer structure than RPIF-15-0, so the flame retardancy of RPIF-10-5 is superior to that of other samples. Meanwhile, the RPIF-5-10 residue’s degree of graphitization is basically equivalent to that of RPIF-10-5, but obvious cracks (Figure 13(d-1)) are found, and RPIF-10-5 has better safety, further confirming the importance of generating an ideal and dense char layer for RPIF to enhance flame retardancy.

Meanwhile, the XPS survey spectra of the RPIF residue are shown in Figure 14c, and the elemental contents are listed in Table S3. In Figure 14c and Table S3, the C/O ratio of RPIF-10-5 is obviously greater than those of the others, and that of RPIF-0-0 is only 3.24, which means that the RIF-10-5 residue possesses the highest proportion of carbon. The char layer with a higher carbon content will form the more effective fire protection layer and hinder the transmission of flame. So, RPIF-10-5 presents the highest flame retardancy. In theory, RPIF-15-0 possesses the highest EG dosage and the residue should have a C/O ratio. Following the comparison presented in this paper, it is speculated that the phosphorous flame-retardant chain segment may play another role. That is, the generation of phosphoric acid derivatives from the pyrolysis of PPCP may also be involved in catalytic dehydration and deoxygenation carbonization in the matrix resin, because the C/O ratio of the RPIF-0-15 residue is also higher than that of RPI-0-0.

**Figure 15:**
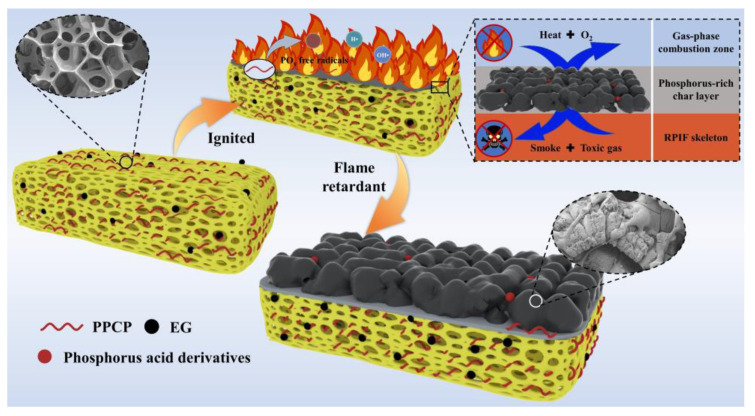
Schematic illustration for flame-retardant and smoke-suppressant effect mechanisms of RPIF.

Figure 14d–f show the fitting analysis of high-resolution P 2p, C 1s, and O 1s XPS spectra of the RPIF-10-5 residue, respectively. The high-resolution P 2p XPS spectra in Figure 15d show three peaks at 132.1 eV, 133.2 eV, and 134.1 eV. They correspond to the P-O-C, O-P=O, and P-C structures, respectively [41]. In addition, deconvolution peaks of C-P and O-P appear in the high-resolution C 1s and O 1s XPS spectra (Figure 14e,f), respectively. The results obtained in this study indicate that the crosslinking phosphorus oxides and phosphorus carbon compounds generated by the thermal decomposition of PPCP are present in the char layer. They can play a chemical crosslinking role for residues and increase the integrity of the carbon layer. Finally, a barrier layer with a denser structure and higher carbon content is obtained, and exerts an excellent flame-retardant effect for RPIF.

Based on the above analysis, the flame-retardant effect, smoke-suppressant, and toxic fume inhibition mechanism schematics are shown in Figure 15. When the surface of the RPIF begins to ignite, EG rapidly expands and forms worm-like graphitic carbon, which can adsorb smoke particles and toxic fume, including HCN and CO. Meanwhile, the introduced PPCP starts to degrade before the matrix resin and releases PO radicals to catch H, HO, and hydrocarbyl radicals in the gas-phase combustion zone, so their combustion reaction with oxygen and combustion degree in the gas-phase are all weakened. These two factors directly enhance the flame retardancy and use safety of RPIF. Going a step further, the phosphoric acid derivatives in the condensed phase play a chemical bonding role; they work together with expanded EG to form a char layer with a higher graphitization degree, more complete surface structure, and a higher C/O ratio. Then, an ideal char layer with refractory and isolation functions is obtained by the combination of the introduced EG and PPCP. This directly decreases the combustion degree of RPIF and enhances its use safety, including flame retardancy and toxic fume generation behavior.

## 4. Conclusions

In this work, reactive PPCP was successfully synthesized to realize chemical bonding between the RPIF matrix and a phosphorous flame-retardant chain segment. This design solves the volatilization problem of common liquid phosphorus-containing flame retardants in the high-temperature curing process of RPIF. The introduction of PPCP alone still leads to serious drawbacks in smoke and toxic fume release, including increases in PSPR, TSP, Ds max, TCOP, and HCN concentration. However, the combinational use of EG can effectively reduce these drawbacks, so EG can be considered as an ideal partner for PPCP. Moreover, the combination of PPCP and EG synergistically enhances the flame retardancy and use safety of RPIF owing to the unique structure of a dense char layer. When the total dosage of additives including EG and PPCP is fixed and they are simultaneously applied to RPIF, a higher EG dosage will about a higher flame retardancy in RPIF; the most preferred ratio of EG and PPCP is 2:1 (RPIF-10-5) in this study. The corresponding RPIF also has very low PHRR and THR, which further shows the effectiveness of the combinational use of EG and PPCP in improving flame retardancy for RPIF. With the combinational use of EG and PPCP, the phosphoric acid derivatives coming from the pyrolysis of PPCP can realize the crosslinking between worm-like graphitic carbon, and then a dense, integral, and porous char layer is formed. Thus, the flame can be effectively obstructed from the matrix resin, the generation of toxic fumes can be effectively decreased, and the generated smoke can also be fully absorbed by the porous char layer. Finally, FT-IR, Raman, and XPS data of char residues further prove the effectiveness of enhancing the use safety of RPIF via a high C/O ratio, graphitization degree, and so on. The obvious positive synergy between flame-retardancy and smoke-suppression in RPIF derived from the combinational use of EG and PPCP effectively improves the use safety of RPIF; this design presents strong advantages for the application of RPIF in the building insulation field.

## Figures and Tables

**Table 1:** Formulation of addition agent of RPIF.

Sample	EG (wt. %)	PPCP (wt. %)	Sample	EG (wt. %)	PPCP (wt. %)
RPIF-0-0	0	0	RPIF-5-5	5	5
RPIF-0-5	0	5	RPIF-10-5	10	5
RPIF-0-10	0	10	RPIF-10-10	10	10
RPIF-0-15	0	15	RPIF-10-15	10	15
RPIF-5-0	5	0	RPIF-5-10	5	10
RPIF-10-0	10	0	RPIF-15-10	15	10
RPIF-15-0	15	0			

## Data Availability

The authors are not permitted to share data.

## Supplementary Materials

The following supporting information can be downloaded at: https://www.mdpi.com/article/10.3390/polym15061381/s1, Figure S1: HRR and THR curves of RPIF-E-0 and RPIF-0-P series; Figure S2: SPR and TSP curves of RPIF-E-0 and RPIF-0-P series; Figure S3: Compressive stress-strain curves of (a) RPIF-E-0 series and (b) RPIF-0-P series; Table S1: TGA data of partial RPIF; Table S2: Properties of RPIF; Table S3: Elemental content of RPIF residues after CCT.
