# Supplementary material for: Fabrication of Rigid Isocyanate-Based Polyimide Foam Achieved Excellent Use Safety via Synergy between Expandable Graphite and Phosphorus-Containing Polyol
Source: Polymers (Basel). 2023 Mar 10;15(6):1381. doi: 10.3390/polym15061381 (PMC10052713; doi:10.3390/polym15061381)
Supplement: Supplementary file 1 [file polymers-15-01381-s001.zip › polymers-2036876-supplementary.pdf]

Supporting information

Fabrication of rigid isocyanate-based polyimide foam with excellent using safety owing to synergy between expandable graphite and phosphorus-containing polyol

Junhe Zhang[a], Yingze Liu[a], Xin Fu[b], Ting Wang[b], Gaohui Sun[a,], Xu Zhang[c], Shihui Han[a,**]*

[a] College of Materials Science and Chemical Engineering, Harbin Engineering University, Harbin 150001, PR China

[b] Wuhan Second Ship Design and Research Institute, Wuhan 430064,PR China

[c] School of Safety Engineering, Shenyang Aerospace University, Shenyang 110136, PR China

Scheme S1. Schematic diagram of RPIF preparation process.

Figure S1. HRR and THR curves of RPIF-E-0 and RPIF-0-P series.

Figure S2. SPR and TSP curves of RPIF-E-0 and RPIF-0-P series.

Table S1. Formulation of addition agent and RPIF density.

Table S2. TGA data of partial RPIF.

Table S3. CCT and Ds results of RPIF.

Table S4. Elemental content of RPIF residues after CCT.

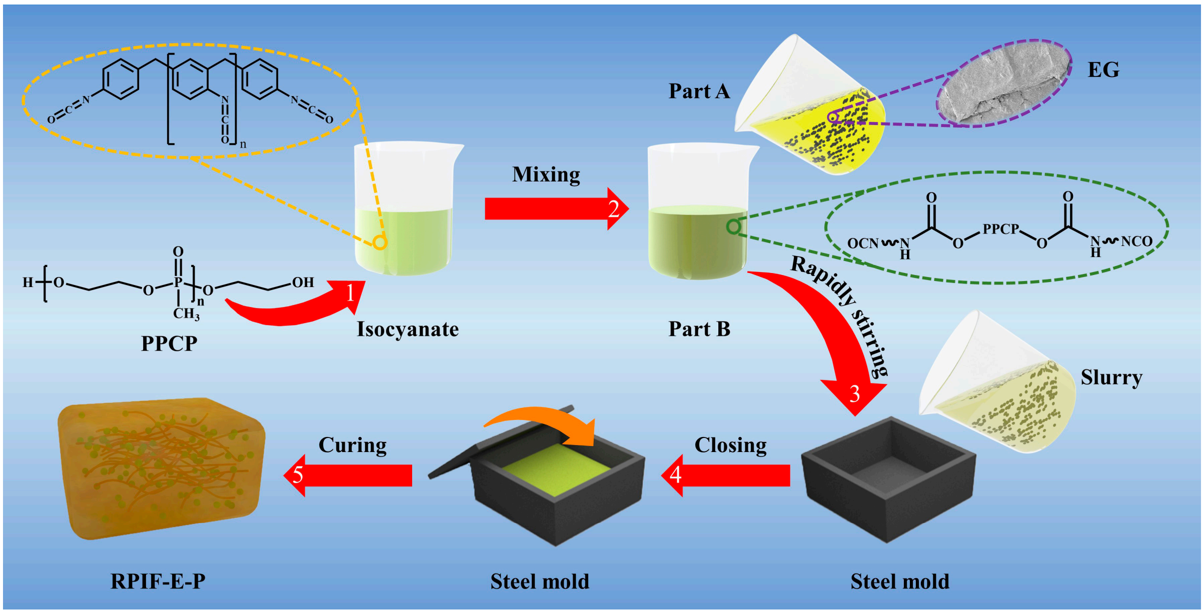

Scheme S1. Schematic diagram of RPIF preparation process.

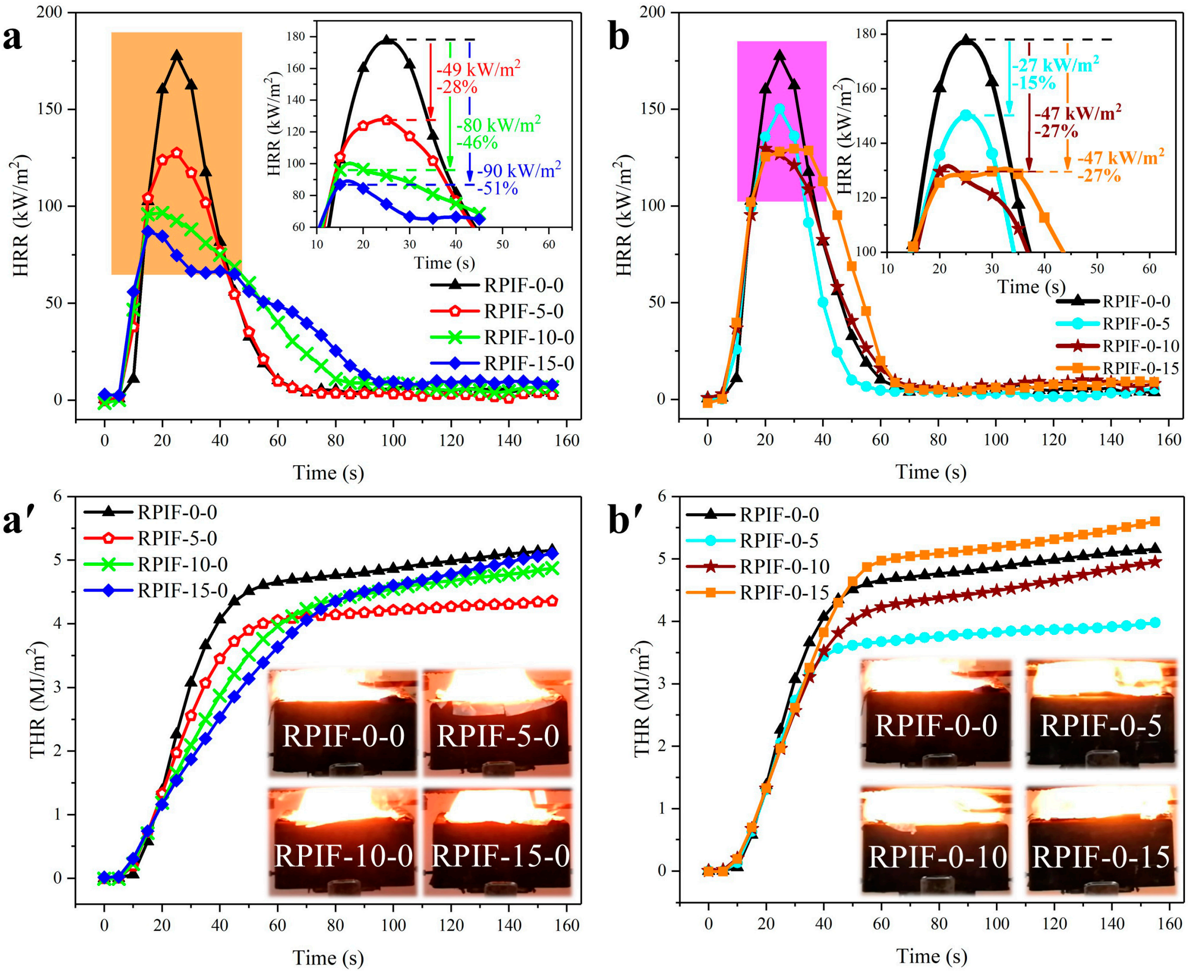

Figure S1. HRR and THR curves of RPIF-E-0 (a, a') series and RPIF-0-P (b, b') series.

For RPIF-E-0 series, HRR and THR curves in Figure S1 decrease significantly with

increased EG dosage. As shown in Table S3, PHRR of RPIF-0-0 reaches to 177 kW/m^2, but that of RPIF-5-0, RPIF-10-0, and RPIF-15-0 decreases to 128 kW/m^2, 97 kW/m^2, and 87 kW/m^2 and corresponds to an increasingly evident reduction at 28%, 46%, and 51% comparing with RPIF-0-0, respectively. The brightness and size of burning flame is also gradually decreasing. What is not ideal is that the THR in this series does not present the similar change as PHRR, the top reduction in THR comparing to RPIF-0-0 is only 16% (RPIF-5-0, 4.36 MJ/m^2), and the reduction of RPIF-10-0 and RPIF-15-0 are lower owing to the appearance of the second heat releasing region in HRR curves. The decrease in PHRR indicates that EG can decrease the burning intensity of RPIF. It attributes to the forming of worm-like graphitic carbon generating from the expansion of EG which can insulate flame. However, because the alone worm-like loose porous graphitic carbon cannot form a dense and continuous carbon layer structure, the heat and oxygen still can gradually pass through carbon layer into the interior resin, this directly leads to the resurgence of combustion and appearance of the second peak of HRR, thus the THR cannot be drastically reduced.

For RPIF-0-P series which only applying reactive PPCP as flame retardant, all HRR curves in Fig. S1b only display the unimodal modality. PHRR decreases with increased PPCP dosage, that of RPIF-0-5, RPIF-0-10, and RPIF-0-15 reduces to 150 kW/m^2, 130 kW/m^2, and 130 kW/m^2, corresponding to a reduction of 15%, 27%, and 27% comparing with RPIF-0-0, respectively. THR of RPIF-0-5 and RPIF-0-10 also presents a reduction of 23% and 4% comparing with RPIF-0-0, respectively. These results indicate that PPCP shows an effective suppression for the severity of burning behavior of RPIF. These mainly owe to the effective quenching of PO radical generated from decomposition of PPCP component in resin for H

radical and hydrocarbyl radical during combustion process which can release amounts of heat accompanying reaction with oxygen.

However, THR of RPIF-0-15 is 0.44 MJ/m^2 higher than that of RPIF-0-0. It is maybe caused by the saturated aliphatic structure in PPCP which can release more heat than aromatic groups, thus the more introduction of PPCP, the higher THR. Meanwhile, these results indicate that EG shows more effective weakening effect for violent combustion behavior of RPIF than PPCP when they are at same dosage, EG play more effective influence in improvement of using safety and fireproof performance of RPIF when they are alone applied.

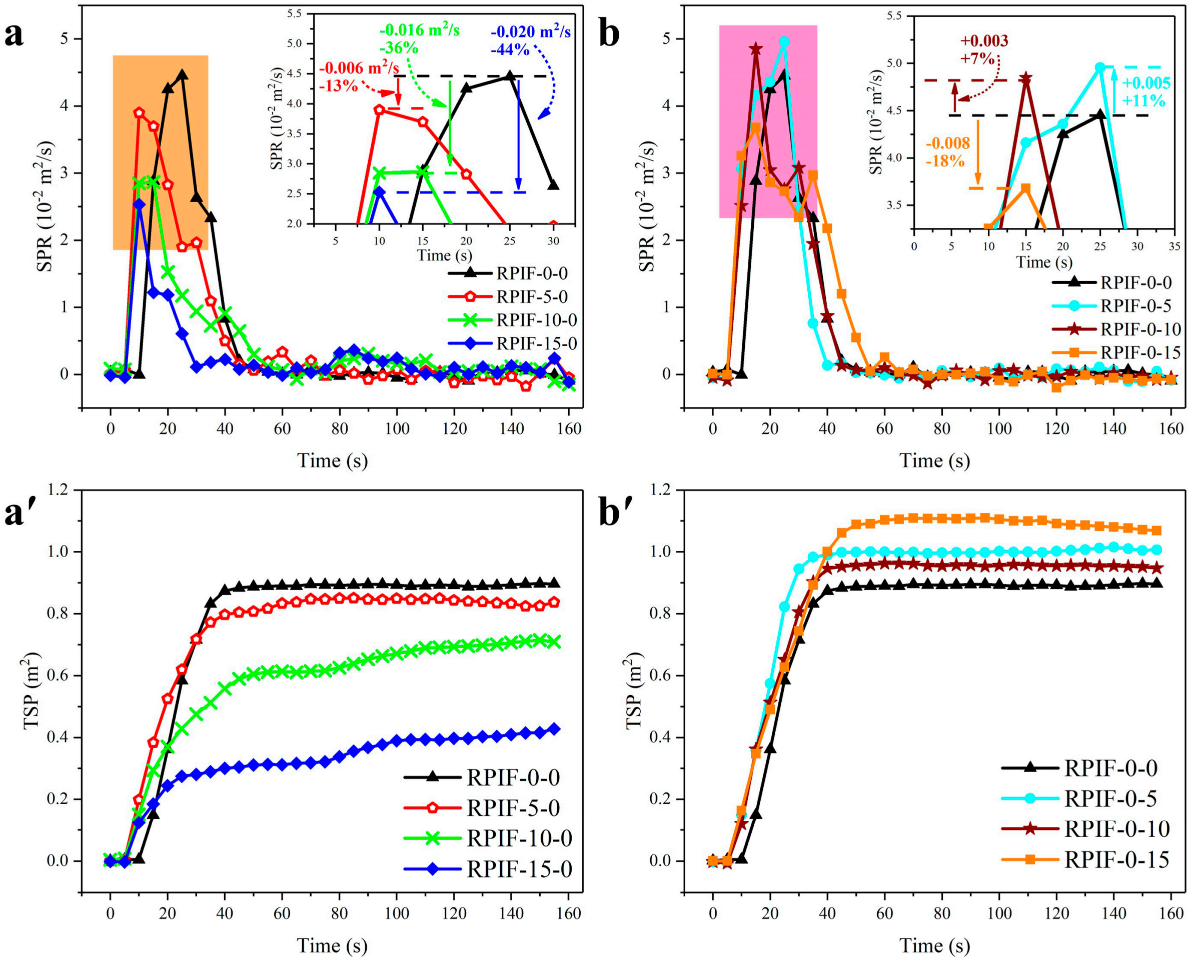

Figure S2. SPR and TSP curves of RPIF-E-0 (a, a') series and RPIF-0-P (b, b') series.

Figure S2 presents SPR and TSP curves of RPIF-E-0 and RPIF-0-P series, detailed

smoke production results including PSPR and TSP at 155 s are listed in Table S3. For RPIF-E-0 series, SPR curve, TSP value at 155 s, and PSPR all decline obviously with increased EG dosage. Referring to the results in Table S3, it detailed shows the change. PSPR of RPIF-0-0 reaches to 0.045 m^2/s, but that of RPIF-5-0, RPIF-10-0, and RPIF-15-0 decreases to 0.039 m^2/s, 0.028 m^2/s, and 0.025 m^2/s, respectively. TSP at 155 s of RPIF-0-0 reaches to 0.90 m^2, but that of RPIF-5-0, RPIF-10-0, and RPIF-15-0 gradually reduces to 0.83 m^2, 0.70 m^2, and 0.42 m^2, they correspond to a minifying of 7.7%, 22.2%, and 53.3%, respectively. These outstanding changes plenty demonstrate the effective smoke suppression of EG on RPIF. It attributes to adsorption effect of porous and worm-like graphitic carbon for particulate matter generated during RPIF combustion. The formation of porous structure is equivalent to the formation of small collectors inside the material to smoke particles, it acts like as a gas mask and smoke can be effectively weakened and adsorbed in initial stage of generation. Moreover, carbon layer structure formed by EG also presents filter action for smoke and directly inhibits the diffusion to environment. In contrast, these results effectively indicate that EG presents more excellent smoke suppression behavior than flame retardancy performance for RPIF.

For RPIF-0-P series, comparing with RPIF-0-0, PSPR of RPIF-0-5 and RPIF-0-10 reaches to 0.050 m^2/s and 0.048 m^2/s, they correspond to an enhancement of 11% and 7%, respectively. However, that of RPIF-0-15 drops to 0.037 m^2/s and corresponds to decrease at 18%. In addition, TSP value at 155s of RPIF-0-5, RPIF-0-10, and RPIF-0-15 increases to 1.01 m^2, 0.95 m^2, and 1.07 m^2, they correspond to an increase of 12%, 6%, and 19% comparing with RPIF-0-0, respectively. The significantly difference from EG is that these results all

demonstrate the alone incorporation of PPCP is adverse for the controlling and weakening of smoke releasing. The alone use of PPCP dramatically increase the smoke toxicity and safety hazard of RPIF during combustion process. Although, use of PPCP effectively enhances fire resistance of RPIF, its alone use is adverse for the improvement in comprehensive using safety. The negative effect of PPCP in smoke releasing behavior owes to its gas-phase flame-retardant process and mechanism. The essence of smoke is solid particles, PO radical generating from pyrolysis of PPCP can capture H, OH, and hydrocarbyl radicals in the gas-phase combustion zone, it greatly promotes the increase in small molecular solid fragments and then leads to the enhanced smoke releasing behavior [32]. The decrease in first PSPR of RPIF-0-15 is maybe caused by the dense char layer owing to increased bonding between carbon network skeleton and crosslinked phosphorus oxide or phosphorus carbon compounds for the enhancement in PPCP dosage. It blocks the smoke releasing path and delays the smoke releasing behavior. However, since there is no effective porous structure, the generated smoke will eventually be completely released. So, the second PSPR appears in SPR curve of RPIF-0-15 and TSP is higher than that of RPIF-0-0.

Table S1. Formulation of addition agent and RPIF density.

Sample	EG (wt%)	PPCP (wt%)	Density (kg/m^3)	Sample	EG (wt%)	PPCP (wt%)	Density (kg/m^3)
RPIF-0-0	0	0	29.34	RPIF-5-5	5	5	32.54
RPIF-0-5	0	5	30.24	RPIF-10-5	10	5	30.34
RPIF-0-10	0	10	31.24	RPIF-10-10	10	10	30.92
RPIF-0-15	0	15	30.47	RPIF-10-15	10	15	32.42
RPIF-5-0	5	0	28.80	RPIF-5-10	5	10	31.46
RPIF-10-0	10	0	31.62	RPIF-15-10	15	10	32.52
RPIF-15-0	15	0	28.44				

Table S2. TGA data of partial RPIF.

Sample	RPIF-0-0	RPIF-15-0	RPIF-0-15	RPIF-5-10	RPIF-10-5
$T_{5\%}$ (°C)	263	257	211	225	230
R_w (%)	32.91	37.69	42.17	41.84	40.83

Table S3. CCT and Ds results of RPIF.

Sample	PHRR (kW/m^2)	THR (MJ/m^2)	PSPR (10^{-2} m^2/s)	TSP (m^2)	Ds max (without flame)	Ds max (with flame)
RPIF-0-0	177	5.16	4.5	0.90	28.43	23.15
RPIF-0-5	150	3.98	5.0	1.01	-	-
RPIF-0-10	130	4.95	4.8	0.95	-	-
RPIF-0-15	130	5.60	3.7	1.07	42.44	30.88
RPIF-5-0	128	4.36	3.9	0.83	-	-
RPIF-10-0	97	4.87	2.8	0.70	-	-
RPIF-15-0	87	5.10	2.5	0.42	14.98	12.82
RPIF-5-5	119	4.68	4.0	0.84	-	-
RPIF-5-10	101	4.13	3.7	0.75	34.44	23.48
RPIF-10-5	85	4.18	2.5	0.60	23.15	15.54

Table S4. Elemental content of RPIF residues after CCT.

Sample	RPIF-15-0	RPIF-0-15	RPIF-5-10	RPIF-10-5
C (%)	81.85	75.75	82.54	84.63
N (%)	5.87	8.59	7.12	6.39
O (%)	12.28	14.59	9.88	8.45
P (%)	-	1.07	0.46	0.53
